# Mapping the cryptic spread of the 2015–2016 global Zika virus epidemic

**DOI:** 10.1186/s12916-020-01845-x

**Published:** 2020-12-17

**Authors:** Haoyang Sun, Borame L. Dickens, Mark Jit, Alex R. Cook, L. Roman Carrasco

**Affiliations:** 1 Saw Swee Hock School of Public Health, National University of Singapore, 12 Science Drive 2, Singapore, 117549 Republic of Singapore; 2 Department of Infectious Disease Epidemiology, London School of Hygiene and Tropical Medicine, Keppel Street, London, WC1E 7HT UK; 3 Modelling and Economics Unit, Public Health England, London, UK; 4 Department of Biological Sciences, National University of Singapore, 14 Science Drive 4, Singapore, 117543 Republic of Singapore

**Keywords:** Zika virus, Epidemic preparedness, Global health, Surveillance capacity, Risk assessment, Undetected transmission, Mathematical modelling

## Abstract

**Background:**

Zika virus (ZIKV) emerged as a global epidemic in 2015–2016 from Latin America with its true geographical extent remaining unclear due to widely presumed underreporting. The identification of locations with potential and unknown spread of ZIKV is a key yet understudied component for outbreak preparedness. Here, we aim to identify locations at a high risk of cryptic ZIKV spread during 2015–2016 to further the understanding of the global ZIKV epidemiology, which is critical for the mitigation of the risk of future epidemics.

**Methods:**

We developed an importation simulation model to estimate the weekly number of ZIKV infections imported in each susceptible spatial unit (i.e. location that did not report any autochthonous Zika cases during 2015–2016), integrating epidemiological, demographic, and travel data as model inputs. Thereafter, a global risk model was applied to estimate the weekly ZIKV transmissibility during 2015–2016 for each location. Finally, we assessed the risk of onward ZIKV spread following importation in each susceptible spatial unit to identify locations with a high potential for cryptic ZIKV spread during 2015–2016.

**Results:**

We have found 24 susceptible spatial units that were likely to have experienced cryptic ZIKV spread during 2015–2016, of which 10 continue to have a high risk estimate within a highly conservative scenario, namely, Luanda in Angola, Banten in Indonesia, Maharashtra in India, Lagos in Nigeria, Taiwan and Guangdong in China, Dakar in Senegal, Maputo in Mozambique, Kinshasa in Congo DRC, and Pool in Congo. Notably, among the 24 susceptible spatial units identified, some have reported their first ZIKV outbreaks since 2017, thus adding to the credibility of our results (derived using 2015–2016 data only).

**Conclusion:**

Our study has provided valuable insights into the potentially high-risk locations for cryptic ZIKV circulation during the 2015–2016 pandemic and has also laid a foundation for future studies that attempt to further narrow this key knowledge gap. Our modelling framework can be adapted to identify areas with likely unknown spread of other emerging vector-borne diseases, which has important implications for public health readiness especially in resource-limited settings.

## Background

Zika virus (ZIKV) is a *flavivirus* that is mainly transmitted by the *Aedes* mosquitoes [1]. Since its first isolation in a Ugandan forest in 1947 [2], the virus has until recent years only caused regional outbreaks [3, 4]. In February 2016, a Public Health Emergency of International Concern was declared by the World Health Organization, as ZIKV swept through the majority of the countries in Latin America and the Caribbean, coinciding with an unusual increase in the number of microcephaly cases and other neurological disorders [5]. The reported ZIKV incidence declined substantially after 2016 [6], but in the meantime, new scientific evidence continued to emerge and reveal new locations where ZIKV circulation had never been identified before [7]. More recently, the first autochthonous ZIKV case in Europe was reported in October 2019 with its source remaining unknown at the time of writing [8], which underscores our limited understanding of the virus's epidemiology. Although ZIKV is no longer a public health emergency, the potential reoccurrence of future large-scale epidemics remains a concern, which necessitates continued investments in ZIKV research and surveillance in preparation for such an event [9].

To date, modelling studies have yielded important insights into the virus's transmission dynamics [10–14], ecological niche [15–17], and at-risk population size [18], but little has been done to understand the gap between where cases may have already occurred and where they have been reported [19]. Due to the high proportion of ZIKV infections that are asymptomatic and its similarity in clinical presentation to other diseases such as dengue fever [3], undetected or unreported spread of ZIKV was widely presumed, especially in countries with limited public health resources [17]. The identification of these locations is critical, as their healthcare systems may be overwhelmed during possible future waves of epidemics if ill prepared, and due to the risk of onward dissemination via air travel. Whilst a recent study used travel surveillance and viral genomes data to detect an unreported ZIKV outbreak in Cuba [20], our understanding of the geographical areas that are likely to have experienced cryptic ZIKV spread is still seriously lacking at a global scale. To narrow this knowledge gap requires a novel modelling framework integrating a wide range of factors that determine the worldwide introduction of the virus and the subsequent autochthonous transmission.

In this study, we aim to answer the following key questions, focusing on all countries or first-level subdivisions where no indigenous Zika cases were reported during the 2015–2016 global epidemic: (i) At least how many ZIKV infections were imported in each country or subdivision during 2015–2016 and to what extent were imported ZIKV infections underreported? (ii) Which countries or subdivisions were most likely to experience cryptic ZIKV spread during the 2015–2016 global epidemic based on currently available data?

## Methods

### Data

#### Epidemiological data

The weekly number of reported autochthonous Zika cases during 2015–2016 for each country in the Americas was published as bar charts by the Pan American Health Organization (PAHO) [21]. These data were digitised by the Andersen Lab for a genomic epidemiological study [22] and made publicly available [23]. We used the Web Plot Digitizer to extract the weekly number of notified Zika cases in Brazil during 2015 published by Lourenço et al. [24], since these data were not published by the PAHO. To achieve a higher spatial resolution, we also obtained weekly cumulative case counts for each first-level subdivision of Colombia between Eweek 38 and Eweek 52 of 2016 from the Colombian National Institute of Health’s website [25]. Each time series was then differenced to derive the weekly case counts and combined with data compiled by Siraj et al. [26], which included weekly notified case data prior to Eweek 38 of 2016. For weekly autochthonous Zika cases reported by countries outside the Americas, we manually reviewed information compiled by HealthMap alongside additional data sources such as ReliefWeb [27, 28] (refer to the Additional File 1: supporting information [29–33] for more details). Each reported case was located to a first-level country subdivision whenever possible, and both confirmed and suspected autochthonous cases were included in our study (Additional File 2: Data S1).

In addition to autochthonous case data, we obtained the reported total number of imported Zika cases during 2015–2016 for each US state, which was published by the Centres for Disease Control and Prevention (collection of imported case data for Florida and Texas was not needed for the study, as discussed in later sections) [34]. For all the other countries or subdivisions that did not report any autochthonous cases during 2015–2016, the imported case data were collected using HealthMap [27].

#### Travel data

We requested the yearly average length of stay (LOS) on inbound tourism trips up to 2016 from the United Nations World Tourism Organization (UNWTO) [35]. For each country, we took the most recently available estimate for the analyses. In most cases, estimates were derived based on the check-in dates from arrival and departure cards or border survey data, but if such data were unavailable for a country, the estimate based on the Hotel Occupancy Survey was used instead [35]. In the rare event that the data were completely missing for a given country, we took the conservative approach and assumed the average LOS to be equal to the minimum value among all the countries under study, to avoid overestimating the number of imported ZIKV infections (refer to section “Simulation of the number of imported infections” for more details).

The monthly number of air ticket bookings during 2015–2016 was obtained from the Official Airline Guide (OAG) for every origin-destination route with up to two connections. This was used to derive the weekly number of bookings assuming the daily number of bookings was uniform within each month for each route. For each first-level country subdivision with no incoming air passengers during 2015–2016, we performed an estimation of the most likely airport (within the same country) that the population would rely on when they returned home (details shown in the Additional File 1: supporting information [36]). The subdivision was subsequently merged with the one where the identified airport was located, and they were modelled as a single unit from then on. For each country where autochthonous cases could not be located to the subdivision level, the entire country was treated as a single unit of analysis. Hence, the spatial unit of analysis in our study can be a single first-level country subdivision, a combination of subdivisions, or an entire country (hereinafter referred to as “spatial unit”). Countries with zero incoming air passenger during 2015–2016 were excluded from the analysis (refer to the Additional File 1: supporting information for more details).

#### Demographic and ecological data

We obtained the global estimated 2015-population counts at a ~ 1 km × 1 km resolution from the Socioeconomic Data and Applications Centre (SEDAC) [37], which was used to derive the total population within each spatial unit. The daily temperature at 2 m between 2015 and 2016 was obtained from the European Centre for Medium-Range Weather Forecasts at ~ 30 km × 30 km resolution [38]. Each temperature map was resampled using bilinear interpolation based on the cell size of the SEDAC population map. Subsequently, the resampled temperature pixel values were averaged within each spatial unit for each day, using the corresponding 2015-population counts as weights. We did not calculate raw average values because the population distribution can be highly uneven for some spatial units such as the Sichuan province of China, where the majority reside within the basin instead of the mountain or plateau regions.

The methods employed to quantify the global environmental suitability of *Ae. aegypti* and *Ae. albopictus*, as well as the associated uncertainties, were described in Dickens et al. [39]. For each species, we obtained 250 vector suitability maps at a ~ 5 km × 5 km resolution via bootstrapping. Our models performed reasonably well based on the out-of-sample prediction accuracy, with a median true skill statistic of 0.84 (0.76–0.86) for *Ae. aegypti* and 0.71 (0.66–0.78) for *Ae. albopictus* [39]. Following the same procedure as above, each vector suitability map was resampled according to the resolution of the SEDAC population map to derive the average suitability value weighted by the human population counts for each spatial unit.

### Statistical analyses

#### Overview

**Fig. 1:**
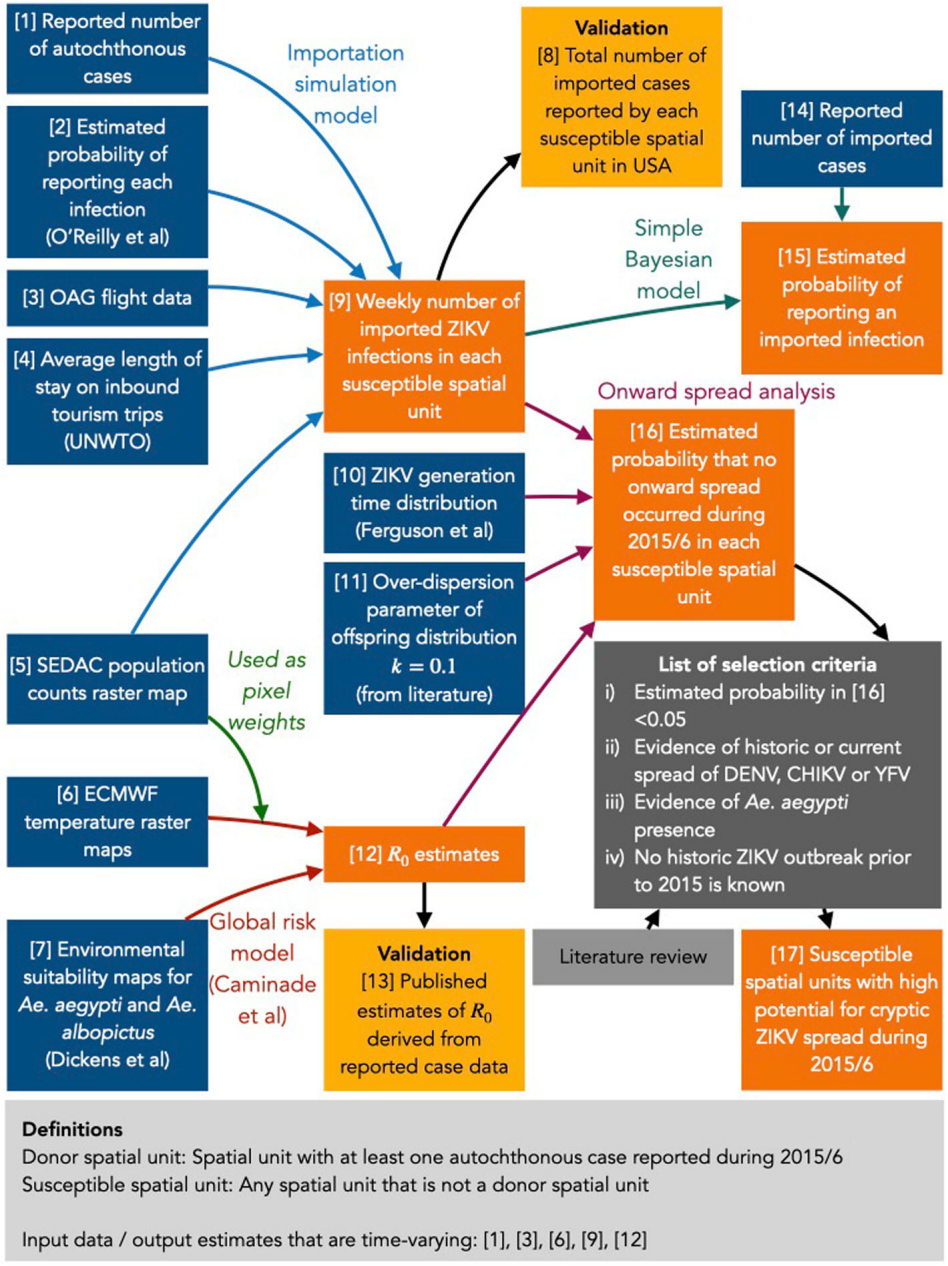
Schematic overview of the methods. Blue boxes denote input data, and dark orange boxes output estimates. Note that in the onward spread analysis, we further imposed thermal restrictions for ZIKV transmission and applied a threshold value for the estimated environmental suitability of *Ae. aegypti* to minimise false positives*.* Refer to the “Methods” section “Susceptible spatial units with cryptic spread of ZIKV” for more details

To map the cryptic spread of ZIKV during the 2015–2016 global epidemic, we first performed simulations to estimate the weekly number of ZIKV infections imported into each spatial unit that did not *report* any autochthonous cases during 2015–2016. Hereinafter, we will refer to these spatial units as *susceptible spatial units*, and the rest as *donor spatial units*. As a by-product of the simulation, a reporting index was derived to quantify the probability of reporting a case per importation for each susceptible spatial unit. Next, we estimated the virus's weekly basic reproduction number (*R*_0_) for each susceptible spatial unit, which was then used to compute the probability that no onward spread following ZIKV importation occurred during 2015–2016. These probability estimates derived from our model, together with the local evidence of *Aedes*-borne disease transmission potential based on the existing literature, were used to identify susceptible spatial units with a high chance of cryptic ZIKV spread during 2015–2016 (refer to Fig. 1 for the schematic overview of methods, and Additional File 3 for the R code).

#### Simulation of the number of imported infections

To simulate the weekly number of imported ZIKV infections for each susceptible spatial unit, we first made the following definitions and assumptions. First, the *local population* of a spatial unit was defined as all who live in that spatial unit regardless of citizenship status. At any point in time, the size of a spatial unit *i*’s local population who were visiting other spatial units was assumed to be approximately equal to the total number of spatial unit *i*’s visitors. Hence, the total number of individuals located in a spatial unit at any point in time can be approximated by the total local population size, which was given by the SEDAC population estimates. Second, we assumed that air travellers with origin airport located in spatial unit *i* and destination airport located in spatial unit *j* were only made up of people belonging to the local population of spatial unit *i* or those of spatial unit *j*. Third, people who acquired ZIKV infection while visiting a spatial unit were assumed to remain asymptomatic by the end of their visits, so that all the autochthonous cases reported by a spatial unit can be assumed to come from its local population. Fourth, for any individual who belonged to the local population of a susceptible spatial unit and visited a donor spatial unit during 2015–2016, no immunity against ZIKV developed prior to his/her visit.

A total of 10,000 simulations were performed, where in each simulation *s* = 1, …, 10, 000, we generated an estimate of the number of ZIKV infections imported from a *donor* spatial unit *i* to a *susceptible* spatial unit *j* during Eweek *t*, denoted by ${M}_{i\to j,t}^{(s)}$. Of these, ${M}_{i\to j,t}^{(s)}\left[i\right]$ belonged to the local population of spatial unit *i* and ${M}_{i\to j,t}^{(s)}\left[j\right]$ the local population of spatial unit *j*, which were estimated separately as follows.

To begin with, we divided the autochthonous case count reported in spatial unit *i* during Eweek *t*, by the country-specific reporting rate based on O’Reilly et al.’s study to obtain the actual number of autochthonous infections *I*_*i*, *t*_ [14]. Here, we used *c*_*i*_ to denote the country to which spatial unit *i* belonged, and ${\rho}_{c_i}$ to denote the percentage of autochthonous infections in *c*_*i*_ that were reported, which was assumed to be time-invariant. In each simulation *s*, the reporting rate estimate ${\rho}_{c_i}^{(s)}$ was drawn from a beta distribution with parameters ${a}_{c_i}$ and ${b}_{c_i}$, under which the 2.5th and 97.5th percentiles of the resulting distribution equal the endpoints of the 95% credible interval produced by O’Reilly et al. [14]. For countries where the reporting rate estimate was unavailable, we assumed the parameters for the reporting rate uncertainty distribution to be equal to those for French Guiana, which had the highest reporting rate estimate among all the countries included in O’Reilly et al.’s study [14]. We considered this as a conservative approach, as it sought to avoid overestimating the weekly number of imported infections *M*_*i* → *j*, *t*_. Overall, countries not included in O’Reilly et al.’s study only accounted for less than 3% of the total number of autochthonous cases reported worldwide during 2015–2016. In the equations below, *U*_*i*, *t*_ refers to the autochthonous case count reported by spatial unit *i* in Eweek *t*, and the same realisation ${\rho}_{c_i}^{(s)}$ applied to all spatial units within country *c*_*i*_ and for all Eweeks within each simulation *s*:

$$ {\rho}_{c_i}^{(s)}\sim Beta\left({a}_{c_i},{b}_{c_i}\right), $$

$$ {I}_{i,t}^{(s)}=\frac{U_{i,t}}{\rho_{c_i}^{(s)}}. $$

Next, of the *v*_*i* → *j*, *t*_ air travellers with origin airport located in spatial unit *i* and destination airport located in spatial unit *j* during Eweek *t*, *v*_*i* → *j*, *t*_[*i*] belonged to the local population of spatial unit *i*. In each simulation *s*, ${v}_{i\to j,t}^{(s)}\left[i\right]$ was drawn from a binomial distribution, with number of Bernoulli trials *v*_*i* → *j*, *t*_, and success probability ${\pi}_{c_i,{c}_j}$, where *c*_*i*_ and *c*_*j*_ referred to the countries to which spatial units *i* and *j* belonged respectively. Whilst for each country the UNWTO provided information on the arrivals of non-resident tourists/visitors at the national borders by country of residence, these data were incomplete and only allowed us to estimate the success probability ${\pi}_{c_i,{c}_j}$ for less than 1.6% of the country pairs in our study. In addition, we did not find country-level indicators such as gross domestic product per capita to be informative for predicting ${\pi}_{c_i,{c}_j}$ based on the very few estimates constructed from the UNWTO data. Hence, in each simulation *s*, ${\pi}_{c_i,{c}_j}^{(s)}$ was drawn from a beta distribution with a substantial amount of variation around 0.5 to capture parameter uncertainty, followed by ${v}_{i\to j,t}^{(s)}\left[i\right]$ that was drawn from a binomial distribution as previously described. We then subtracted ${v}_{i\to j,t}^{(s)}\left[i\right]$ from *v*_*i* → *j*, *t*_ to obtain ${v}_{i\to j,t}^{(s)}\left[j\right]$:

$$ {\pi}_{c_i,{c}_j}^{(s)}\sim Beta\left(10,10\right), $$

$$ {v}_{i\to j,t}^{(s)}\left[i\right]\sim Bin\left({v}_{i\to j,t},{\pi}_{c_i,{c}_j}^{(s)}\right), $$

$$ {v}_{i\to j,t}^{(s)}\left[j\right]={v}_{i\to j,t}-{v}_{i\to j,t}^{(s)}\left[i\right]. $$

It should be noted that within each simulation *s*, the realisation ${\pi}_{c_i,{c}_j}^{(s)}$ also applied to all other routes with the same origin and destination countries. In other words, given a pair of origin and destination countries, the aforementioned probability value was generated only once in each simulation.

Therefore, in simulation *s*, the first component of ${M}_{i\to j,t}^{(s)}$ was given by:

$$ {M}_{i\to j,t}^{(s)}\left[i\right]\sim Bin\left({v}_{i\to j,t}^{(s)}\left[i\right],\frac{I_{i,t}^{(s)}}{POP_i-\sum \limits_{k\ne i}{v}_{i\to k,t}^{(s)}\left[k\right]\bullet {LOS}_{c_i}}\right). $$

The justification for the above calculation was as follows. In the denominator, *POP*_*i*_ denoted the total number of people located in spatial unit *i* at Eweek *t* (or any point in time, as previously discussed). Here, we assumed a stable system in which the weekly arrival rate of visitors from the local population of any spatial unit *k* ≠ *i* was equal to the rate at which they exited spatial unit *i*, *v*_*i* → *k*, *t*_[*k*], for any Eweek *t*. On average, these individuals stayed in spatial unit *i* for ${LOS}_{c_i}$ weeks during their visits based on the UNWTO’s estimates. Applying Little’s law originally developed in queueing theory [40], we multiplied $\sum \limits_{k\ne i}{v}_{i\to k,t}^{(s)}\left[k\right]$ by ${LOS}_{c_i}$ in each simulation *s* to obtain the estimated total number of people located in spatial unit *i* at Eweek *t* who did not belong to the local population of spatial unit *i*, which was then subtracted from *POP*_*i*_. Therefore, ${M}_{i\to j,t}^{(s)}\left[i\right]$ can be considered as a realisation of a random variable following a hypergeometric distribution, where ${v}_{i\to j,t}^{(s)}\left[i\right]$ random draws were obtained without replacement from a total number of $\left\{{POP}_i-\sum \limits_{k\ne i}{v}_{i\to k,t}^{(s)}\left[k\right]\bullet {LOS}_{c_i}\right\}$ individuals, of whom ${I}_{i,t}^{(s)}$ carried ZIKV. Here, to ease the computation, we approximated ${M}_{i\to j,t}^{(s)}\left[i\right]$ by drawing from a binomial distribution instead. Note that we only included ${I}_{i,t}^{(s)}$ into the numerator of the success probability, since most autochthonous infections occurring prior to Eweek *t* had recovered by Eweek *t*, given a recovery rate of around 1/7–1/5 per day [11, 13]. In addition, the aforementioned ${I}_{i,t}^{(s)}$ individuals were presumably still able to travel because the vast majority were unreported and likely to be asymptomatic.

Of the total (unobserved) number of incident autochthonous infections occurring in spatial unit *i* during Eweek *t* (denoted by *L*_*i*, *t*_), *M*_*i* → *j*, *t*_[*j*] were visitors belonging to the local population of spatial unit *j*. Conditioning on *L*_*i*, *t*_, *M*_*i* → *j*, *t*_[*j*] followed a binomial distribution with the number of Bernoulli trials *L*_*i*, *t*_ and a success probability as a function of *i*, *j*, and *t*. Since *L*_*i*, *t*_ was reasonably large (i.e. greater than 20 in most cases), we modelled *M*_*i* → *j*, *t*_[*j*] as a Poisson random variable with mean parameter proportional to the total person-time at risk of the visitors from spatial unit *j*, and similarly for the number of autochthonous infections occurring in spatial unit *i* during Eweek *t* that belonged to the local population of spatial unit *i* (*I*_*i*, *t*_). Hence, the ratio between their expected values was given by:

$$ \frac{\mathbbm{E}\left\{{M}_{i\to j,t}\left[j\right]\right\}}{\mathbbm{E}\left\{{I}_{i,t}\right\}}\cong \frac{v_{i\to j,t}\left[j\right]\bullet {LOS}_{c_i}}{\left({POP}_i-\sum \limits_{k\ne i}{v}_{i\to k,t}\left[k\right]\bullet {LOS}_{c_i}\right)\left(1-{\eta}_{i,t}\right)}.\kern1em \left(\ast \right) $$

At any point in time during Eweek *t*, the number of people located in spatial unit *i* was *POP*_*i*_, of whom $\left({v}_{i\to j,t}\left[j\right]\bullet {LOS}_{c_i}\right)$ were visitors from spatial unit *j* by Little’s law [40], and likewise $\left({POP}_i-\sum \limits_{k\ne i}{v}_{i\to k,t}\left[k\right]\bullet {LOS}_{c_i}\right)$ belonged to the local population of spatial unit *i* as derived earlier. In the denominator of equation (∗), we included an additional factor (1 − *η*_*i*, *t*_), which denoted the percentage of the local population of spatial unit *i* that were still susceptible to ZIKV infection in Eweek *t* (i.e. ${\eta}_{i,t}=\sum \limits_{t^{\prime }<t}{I}_{i,t\prime }/{POP}_i$, assuming protective immunity to last at least until the end of our study period after primary ZIKV infection).

Hence, in each simulation *s*, given the “observed data” ${I}_{i,t}^{(s)}$, it can be shown that the posterior predictive distribution of ${M}_{i\to j,t}^{(s)}\left[j\right]$ was as follows, if we impose a uniform prior over the positive real line for $\mathbbm{E}\left\{{I}_{i,t}\right\}$:

$$ {M}_{i\to j,t}^{(s)}\left[j\right]\mid {I}_{i,t}^{(s)}\sim NB\left({I}_{i,t}^{(s)}+1,{\left[{\left(\frac{\mathbbm{E}\left\{{M}_{i\to j,t}\left[j\right]\right\}}{\mathbbm{E}\left\{{I}_{i,t}\right\}}\right)}^{(s)}+1\right]}^{-1}\right). $$

In other words, ${M}_{i\to j,t}^{(s)}\left[j\right]$ was drawn from a negative binomial distribution that modelled the number of failures before the ${\left({I}_{i,t}^{(s)}+1\right)}^{\mathrm{th}}$ success in a sequence of independent Bernoulli trials with equal success probability ${\left[{\left(\frac{\mathbbm{E}\left\{{M}_{i\to j,t}\left[j\right]\right\}}{\mathbbm{E}\left\{{I}_{i,t}\right\}}\right)}^{(s)}+1\right]}^{-1}$, where we derived ${\left(\frac{\mathbbm{E}\left\{{M}_{i\to j,t}\left[j\right]\right\}}{\mathbbm{E}\left\{{I}_{i,t}\right\}}\right)}^{(s)}$ by plugging the previously simulated values ${\left\{{v}_{i\to k,t}^{(s)}\left[k\right]\right\}}_{k\ne i}$ and ${\left\{{I}_{i,{t}^{\prime}}^{(s)}\right\}}_{t^{\prime }<t}$ into equation (*).

Finally, the estimated weekly number of ZIKV infections imported from a *donor* spatial unit *i* to a *susceptible* spatial unit *j* in simulation *s* was given by the sum of its two components. Note that we treated the ${M}_{i\to j,t}^{(s)}\left[j\right]$ individuals as imported infections in Eweek *t* despite the possibility that a certain percentage may have returned to spatial unit *j* slightly after Eweek *t*, because no information was available regarding the distribution of LOS. Given that the average LOS was very short in general, the effect of this decision upon the subsequent analyses of the onward ZIKV spread for each susceptible spatial unit was negligible:

$$ {M}_{i\to j,t}^{(s)}={M}_{i\to j,t}^{(s)}\left[i\right]+{M}_{i\to j,t}^{(s)}\left[j\right]. $$

To validate our importation simulation model, we plotted the median estimate of the total number of ZIKV infections imported in each susceptible spatial unit in the US in 2015–2016 against the corresponding reported case count, both on a log_10_ scale. If our simulation results were reasonably accurate and the reporting rates were similar among these susceptible spatial units, we would expect to see all the data points to be close to a straight line with a slope of one.

#### Reporting index

As a by-product of the importation simulation model, a reporting index was derived to quantify the probability of reporting a case per imported infection for each susceptible spatial unit. *Only within this section* did we further merge all the susceptible spatial units of a country to be analysed as a single unit, provided the reported *imported* cases could not be located to the subdivision level.

For each susceptible spatial unit *j*, we denoted the actual total number of imported ZIKV infections during 2015–2016 as *n*_*j*_, where its uncertainty distribution can be approximated by $\sum \limits_{i,t}{M}_{i\to j,t}^{(s)}\ \left(s=1,\dots, 10000\right)$ as obtained from the importation simulation model. The reporting index *p*_*j*_ was assigned an uninformative prior *Beta*(1, 1) and assumed to be independent from variable *n*_*j*_. Thus, given a specific value of *n*_*j*_, the reported imported case count *x*_*j*_ followed a beta-binomial distribution with uniform density:

$$ f\left({x}_j|{n}_j\right)=\frac{1}{n_j+1},\mathrm{where}\ {x}_j=0,\dots, {n}_j. $$

Using Bayes’ theorem, we can compute *f*(*n*_*j*_| *x*_*j*_) as follows, where *I*(∙) was the indicator function:

$$ f\left({n}_j|{x}_j\right)=\frac{f\left({n}_j\right)f\left({x}_j|{n}_j\right)}{f\left({x}_j\right)}\propto f\left({n}_j\right)\bullet \frac{1}{n_j+1}\bullet I\left({n}_j\ge {x}_j\right). $$

The posterior density of *p*_*j*_ was given by the equation below:

$$ f\left({p}_j|{x}_j\right)=\int f\left({n}_j|{x}_j\right)\bullet f\left({p}_j|{n}_j,{x}_j\right)\ d{n}_j. $$

Hence, for each susceptible spatial unit *j*, we generated 10,000 values from the posterior distribution of the reporting index. Specifically, for *s*^∗^ = 1, …, 10, 000:

i. Draw ${n}_j^{\left({s}^{\ast}\right)}\mid {x}_j$ from the set of simulated numbers of imported infections $\sum \limits_{i,t}{M}_{i\to j,t}^{(s)}\ \left(s=1,\dots, 10,000\right)$, with probabilities proportional to the weights $\frac{1}{\sum \limits_{i,t}{M}_{i\to j,t}^{(s)}+1}\bullet I\left(\sum \limits_{i,t}{M}_{i\to j,t}^{(s)}\ge {x}_j\right)$.
ii. Draw ${p}_j^{\left({s}^{\ast}\right)}\mid {n}_j^{\left({s}^{\ast}\right)},{x}_j$ from $Beta\left({x}_j+1,{n}_j^{\left({s}^{\ast}\right)}-{x}_j+1\right)$.

It should be noted that we found a total of four countries that contained at least one donor spatial unit and one susceptible spatial unit simultaneously. For these countries, we estimated that only an average of 0.47 infections per susceptible spatial unit belonged to “within-country importation”, with New York having the largest number (6.40, which was less than 0.03% of its estimated total number of imported ZIKV infections). Hence, this did not affect the estimation of reporting index, where the reported case data presumably only included Zika cases imported from abroad.

#### Estimation of basic reproduction number

We applied a global risk model developed by Caminade et al. to estimate the daily *R*_0_ of ZIKV within each spatial unit [13], which was subsequently averaged across each Eweek to be used for the analysis of onward ZIKV spread in the section “Susceptible spatial units with cryptic spread of ZIKV”. The model inputs included the human recovery rate (*r*), as well as a set of species-specific parameters: biting rates (*h*_1_, *h*_2_), vector preferences (*ϕ*_1_, *ϕ*_2_), vector-to-host and host-to-vector transmission probabilities (${\uptau}_1^{V\to H}$, ${\uptau}_2^{V\to H}$ and ${\uptau}_1^{H\to V}$, ${\uptau}_2^{H\to V}$), mortality rates (*μ*_1_, *μ*_2_), extrinsic incubation rates (*ν*_1_, *ν*_2_), and vector-to-host ratios (*m*_1_, *m*_2_) [13], where the subscripts 1 and 2 referred to *Ae. aegypti* and *Ae. albopictus* respectively (Table 1). The expected number of secondary *human* ZIKV infections generated by a primary human infection in a fully susceptible population was given by:

$$ {R}_0=\sum \limits_{q=1}^2\left(\frac{\uptau_q^{V\to H}{\uptau}_q^{H\to V}{h}_q^2}{\mu_q(T)}\right)\left(\frac{\nu_q(T)}{\nu_q(T)+{\mu}_q(T)}\right)\left(\frac{\phi_q^2{m}_q}{r}\right). $$

**Table 1:** Parameters for the global risk model. Subscripts 1 and 2 referred to *Ae. aegypti* and *Ae. albopictus* respectively. Unless otherwise stated, parameter values were calculated following Caminade et al. [13]

Symbol	Description	Calculation
*h*_1_, *h*_2_	Biting rates (per day)^#^	*h*_1_ = 0.67, [41] *h*_2_ = *h*_1_/2.
*ϕ*_1_, *ϕ*_2_	Vector preferences	*ϕ*_1_ = 1, *ϕ*_2_ = *ϕ*_1_/2.
${\uptau}_1^{V\to H}$, ${\uptau}_2^{V\to H}$	Vector-to-host transmission probability	${\uptau}_1^{V\to H}=0.5$, ${\uptau}_2^{V\to H}={\uptau}_1^{V\to H}.$
${\uptau}_1^{H\to V}$, ${\uptau}_2^{H\to V}$	Host-to-vector transmission probability	${\uptau}_1^{H\to V}=0.1$, ${\uptau}_2^{H\to V}=0.33{\uptau}_1^{H\to V}.$
*μ*_1_, *μ*_2_	Mortality rates (per day)	${\mu}_1=\frac{1}{1.22+\mathit{\exp}\left(-3.05+0.72T\right)}+0.196,\left(T<22{}^{\circ}C\right)$ ${\mu}_1=\frac{1}{1.14+\mathit{\exp}\left(51.4-1.3T\right)}+0.196,\left(T\ge 22{}^{\circ}C\right)$ ${\mu}_2=\frac{1}{1.1+\mathit{\exp}\left(-4.04+0.576T\right)}+0.11883,\left(T<15{}^{\circ}C\right)$ *μ*_2_ = 0.000339*T*^2^ − 0.0189*T* + 0.336, (15 ° *C* ≤ *T* < 26.3 ° *C*) ${\mu}_2=\frac{1}{1.065+\mathit{\exp}\left(32.2-0.92T\right)}+0.073079.\left(T\ge 26.3{}^{\circ}C\right)$
*ν*_1_, *ν*_2_	Extrinsic incubation rates (per day)	${\nu}_1=\frac{1}{4+\mathit{\exp}\left(5.15-0.123\ T\right)},$ *ν*_2_ = *ν*_1_/1.03.
*m*_1_, *m*_2_	Vector-to-host ratios	Assumed to be proportional to the vector suitability values. The scaling factor was re-calibrated using the *R*_0_ estimate produced by Zhang et al. [11].
*r*	Human recovery rate (per day)	*r* = 1/7.

^#^Given the relatively minor contribution of the biting rates’ temperature-dependence to the variation in *R*_0_ [42], here we treated the biting rates as constant, and the uncertainties in $\frac{h^2{\phi}^2{\tau}^{V\to H}{\tau}^{H\to V}}{r}$ were absorbed by re-calibrating the scaling factor that converted vector suitability values to vector-to-host ratios

We followed Caminade et al. [13] and estimated vector mortality rates and extrinsic incubation rates as a function of the population-weighted average of temperature for each spatial unit and each day. We re-calibrated the scaling factor that transformed vector suitability values to vector-to-host ratios, so that our median *R*_0_ estimate at the temperature 25 °C in French Polynesia would equal to that obtained by Zhang et al. via the fitting of a deterministic model to the 2013 French Polynesia outbreak data [11]. All the other parameters were assumed to be constant. Despite the uncertainty in these parameter values, the factor $\frac{h_1^2{\phi}_1^2{\uptau}_1^{V\to H}{\uptau}_1^{H\to V}}{r}$ can be viewed as a single constant whose uncertainty was absorbed by the aforementioned model re-calibration (Similarly for *Ae. albopictus*, assuming the estimated ratio between the two species for each of the model parameters *h*, *ϕ*, *τ*^*V* → *H*^, and *τ*^*H* → *V*^ in Table 1 to be reasonably accurate).

Instead of producing a single daily *R*_0_ estimate for each spatial unit, we generated 250 values of *R*_0_ per spatial unit and per day, where each value was based on a bootstrap estimate of the population-weighted average of vector suitability within the corresponding spatial unit. To validate the model, we took published estimates of *R*_0_ and compared them with our model estimates to assess the agreement (refer to the Additional File 1: supporting information [41] for the inclusion and exclusion criteria for the published *R*_0_ estimates used for the model validation) [24, 33, 43, 44].

#### Susceptible spatial units with cryptic spread of ZIKV

We calculated the probability that no onward spread of ZIKV occurred following importation during 2015–2016, for each susceptible spatial unit with at least one imported infection throughout the 10,000 simulations. Specifically, in each iteration *s* = 1, …, 10000, we first computed the weekly effective number of imported infections for each susceptible spatial unit *j*, as defined by Perkins et al. [45]. This accounted for the fact that the generation time of ZIKV can be much longer than that of directly transmitted viruses, and hence, each human ZIKV infection can be caused by an earlier infection that had occurred one to five weeks before [45].

$$ {\omega}_u=\frac{1}{7}\underset{7\left(u-1\right)}{\overset{7u}{\int }}\left\{{F}_{ZIKV}\left(x+7\right)-{F}_{ZIKV}(x)\right\} dx, $$

$$ \overset{\sim }{M_{\bullet \to j,t}^{(s)}}=\sum \limits_{u=1}^5{\delta}_j\left[t-u,t\right]\bullet {\omega}_u\bullet {M}_{\bullet \to j,t-u}^{(s)}. $$

In the first equation, the cumulative distribution function of the ZIKV generation time was denoted by *F*_*ZIKV*_. This was estimated by Ferguson et al. and subsequently used by Harris et al. to obtain the weight parameters *ω*_*u*_ ’s [10, 46]. In the second equation, ${M}_{\bullet \to j,t-u}^{(s)}$ referred to the number of ZIKV infections imported in spatial unit *j* during Eweek (*t* − *u*) in simulation *s*, summed over all the donor spatial units. The extra factor *δ*_*j*_[*t* − *u*, *t*] was defined to be one if the weekly mean temperature in spatial unit *j* remained within the thermal range for ZIKV transmission from Eweeks (*t* − *u*) to *t*, and zero otherwise. In other words, sustained temperature suitability for ZIKV transmission was required from the time of each imported infection to its secondary human infection. Here, we used Tesla et al.’s thermal range estimate (22.7–34.7 °C), which took into account the temperature constraints for different stages of mosquito development and ZIKV transmission and has been validated using Zika case data reported by different municipalities in Colombia [47]. We additionally created a highly conservative scenario where a much narrower interval (26–29 °C) was considered based on Mordecai et al.’s estimated temperature range for maximal ZIKV transmission [48], to identify susceptible spatial units with a particularly high likelihood of cryptic ZIKV spread.

The analysis of onward ZIKV spread presented below was largely inspired from Churcher et al. [49]. If we assumed the entire local population to be immunologically naïve, the number of infections generated in Eweek *t* by the $\overset{\sim }{M_{\bullet \to j,t}^{(s)}}$ imported infections followed a negative binomial distribution with expectation $\overset{\sim }{\ {M}_{\bullet \to j,t}^{(s)}}\bullet {R_0}_{j,t}^{\left(\left(s-1\right)\%250+1\right)}\bullet I\left[{aeg}_j^{\left(\left(s-1\right)\%250+1\right)}\ge 0.24\right]$ and size $\overset{\sim }{\ {M}_{\bullet \to j,t}^{(s)}}\bullet k$. Here, we additionally required the bootstrap estimate of the environmental suitability for *Ae. aegypti* (i.e. the main vector for ZIKV) in spatial unit *j*, denoted by ${aeg}_j^{\left(\left(s-1\right)\%250+1\right)}$, to be at least 0.24. This threshold was derived by Dickens et al. that maximised the true skill statistic, which can be used to convert suitability value into binary species presence/absence [39]. The parameter *k* referred to the over-dispersion parameter of the offspring distribution, and a smaller value would lead to a larger variation in the number of secondary infections produced by each primary infection, and a higher chance of zero offspring. Due to the unavailability of the over-dispersion parameter estimates derived from Zika case data, we took a conservative approach and set *k* to be 0.1 (i.e. highly over-dispersed offspring distribution) following previous work by Grubaugh et al. [22], to minimise false positives in our final results. In each iteration *s* = 1, …, 10, 000, we derived the probability that no onward spread of ZIKV occurred following importation in spatial unit *j* during 2015–2016, denoted by ${\theta}_j^{(s)}$. These 10,000 probability values were then averaged to obtain $\overline{\theta_j}$, representing the probability of no onward ZIKV spread in spatial unit *j* averaged over the uncertainty in the weekly numbers of imported infections and local ZIKV transmissibility:

$$ {\theta}_j^{(s)}=\prod \limits_t{\left(1+\frac{{R_0}_{j,t}^{\left(\left(s-1\right)\%250+1\right)}\bullet I\left[{aeg}_j^{\left(\left(s-1\right)\%250+1\right)}\ge 0.24\right]}{k}\right)}^{\overset{\sim }{-{M}_{\bullet \to j,t}^{(s)}}\bullet k}, $$

$$ \overline{\theta_j}=\frac{1}{10,000}\sum \limits_{s=1}^{10,000}{\theta}_j^{(s)}. $$

It should be noted that in the above calculation, we ignored ZIKV immunity among the local population of susceptible spatial unit *j* accumulated via weekly ZIKV importation. This was because the number of imported ZIKV infections $\sum \limits_{i,t}{M}_{i\to j,t}\left[j\right]$ over 2015–2016 was estimated to account for at most ~ 0.03% of the entire local population among all the susceptible spatial units *j*, and hence was negligible.

Under each of the thermal restrictions previously specified, we obtained a list of susceptible spatial units with a high chance of cryptic ZIKV spread during 2015–2016 through the following steps. First, we only retained all the susceptible spatial units with $\overline{\theta}$ below 0.05, and considered any $\overline{\theta}$ values below $0.05/\mid \left\{\overline{\theta}\right\}\mid$ as highly significant findings (provided the spatial unit was also retained in the subsequent filtering process). The calculation of $0.05/\mid \left\{\overline{\theta}\right\}\mid$ can be considered as a Bonferroni correction to control for the type I error, where $\mid \left\{\overline{\theta}\right\}\mid$ referred to the total number of spatial units for which $\overline{\theta}$ was computed. Next, we conducted a literature review to remove any remaining susceptible spatial units where no evidence of historical or current spread of dengue, Chikungunya, or yellow fever was found, as these spatial units were assumed to have very limited potential for ZIKV transmission. Susceptible spatial units without evidence of *Ae. aegypti* establishment based on the existing literature were also excluded even if *Ae. albopictus* was present, since the former is known to be the primary vector for ZIKV transmission.

Finally, we additionally excluded French Polynesia, where a large proportion of the local population had developed immunity against ZIKV due to a large outbreak there in 2013 [50], as our model implicitly assumed the ZIKV seroprevalence for each susceptible spatial unit to be zero at the start of 2015. Any remaining susceptible spatial units with evidence of historical ZIKV transmission but no outbreaks were intentionally retained, since the population-level immune landscape at the beginning of 2015 in these spatial units was still poorly understood in general due to incomplete or outdated serological data.

## Results

### Model validation results

**Fig. 2:**
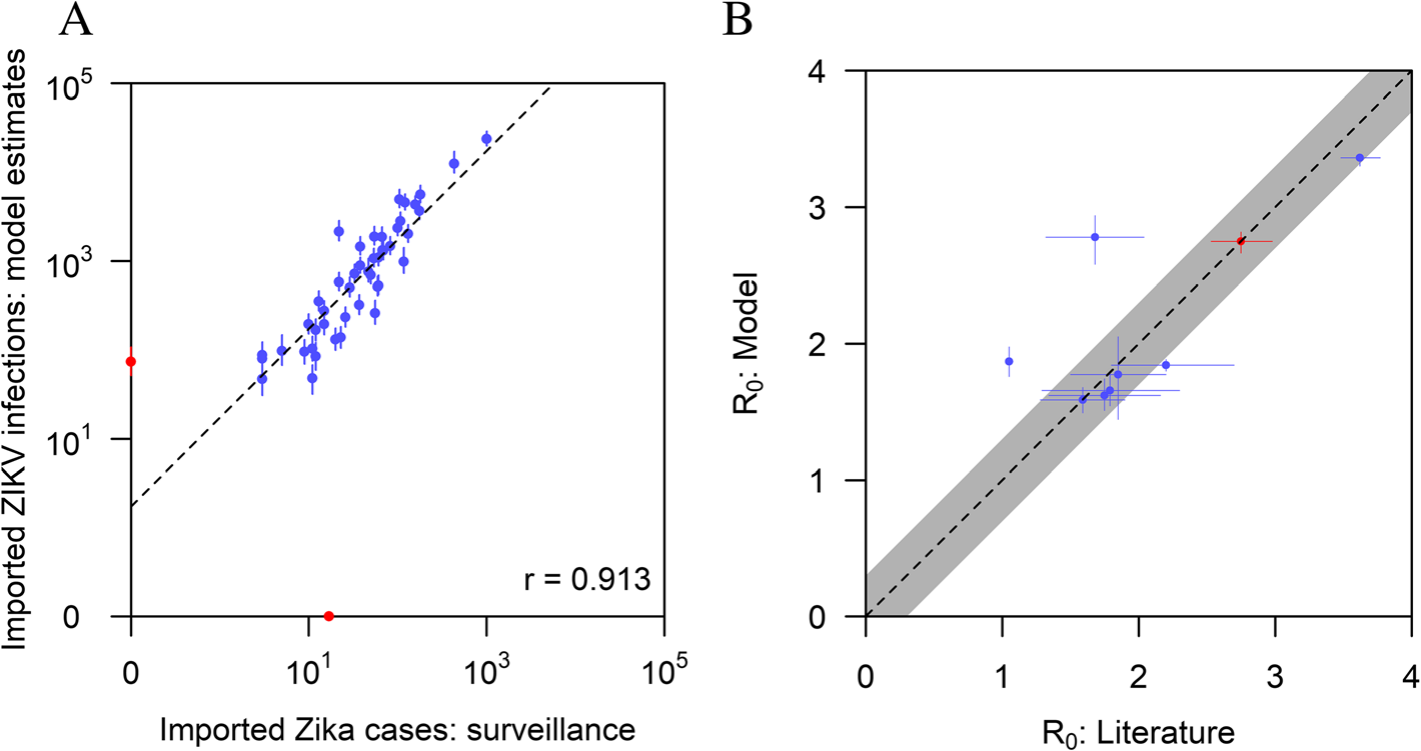
Model validation results. **a** Validation of the importation simulation model. The dashed line was based on a linear regression model with the slope fixed to be one fitted to all observations on a log_10_ scale excluding the two red dots, which show that the reported or the estimated value was zero. **b** Comparison of *R*_0_ values obtained from our estimation versus the existing literature. The grey polygon denotes the ± 0.3 error band and the red dot refers to the *R*_0_ estimate at the temperature 25 °C in French Polynesia by Zhang et al. [11], which was used to calibrate the model

We observed a high correlation between the estimated total number of imported ZIKV infections and the reported case count during 2015–2016 for each susceptible spatial unit in the US (Pearson *r* = 0.913 for log_10_ transformed data). Except two data points, where either the reported or the estimated value was zero, the rest of the observations were close to a straight line with a slope of one (Fig. 2a), suggesting our simulation results were reasonably accurate provided the reporting rates were not drastically different among these susceptible spatial units within the same country. In addition, there was overall a good agreement between the median *R*_0_ estimates derived from the global risk model and those obtained from the literature, with the majority falling within the ±0.3 error band (Fig. 2b). Refer to Additional File 4: Fig. S1 and Additional File 5: Fig. S2 for visualisations of the global risk model outputs.

### Estimated number of imported ZIKV infections and reporting index

**Fig. 3:**
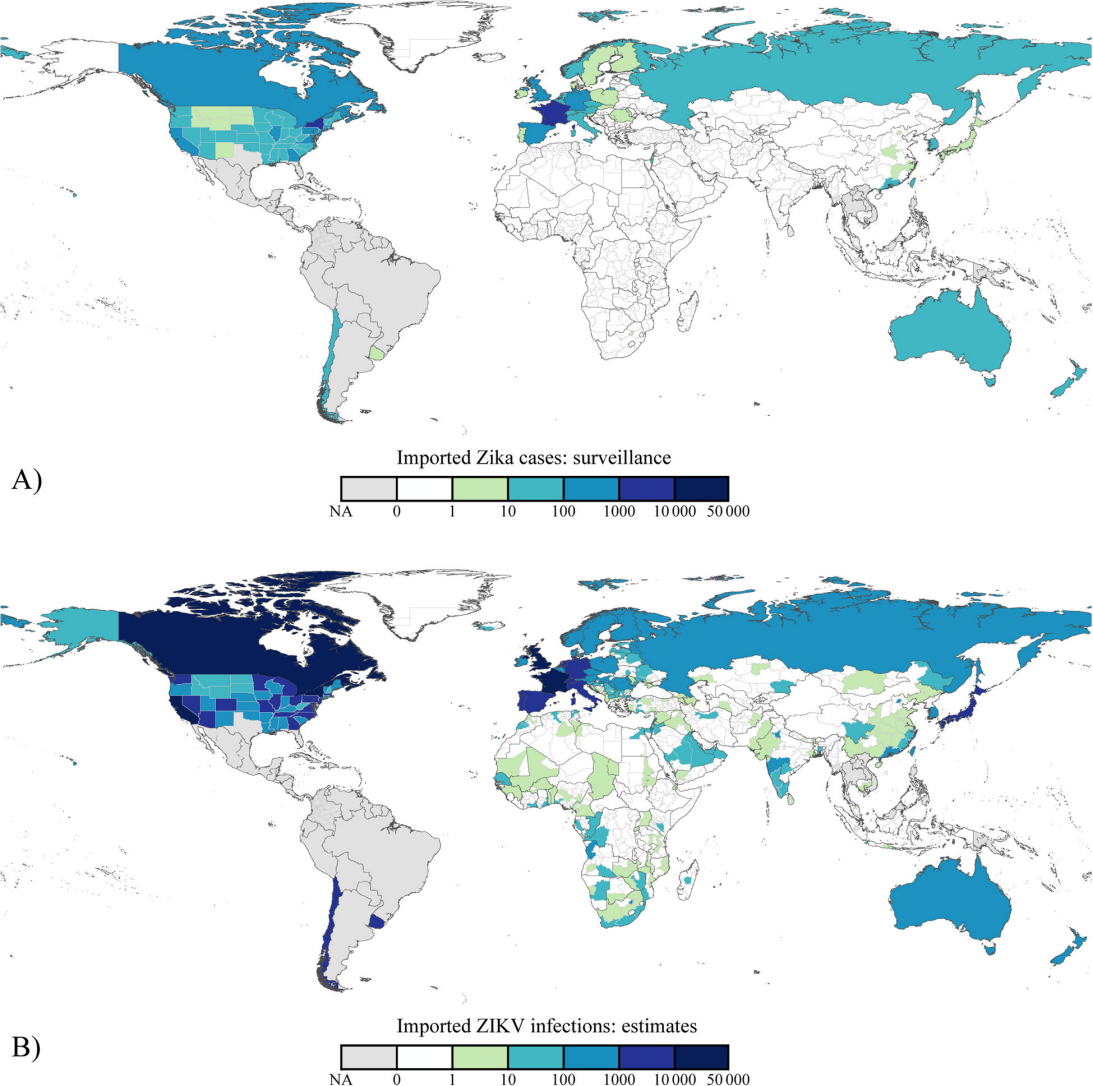
ZIKV importation in each susceptible spatial unit during 2015–2016: **a** Reported case count vs **b** median estimate of the total number of imported infections derived from the simulation model. Each country was treated as a single unit if the imported case count was reported at a country level (e.g. Australia, New Zealand), to facilitate comparison of the two maps. All donor spatial units were coloured in grey

The majority of the susceptible spatial units in Africa and Asia presented relatively low numbers of imported ZIKV infections based on our simulations, which were generally consistent with the reported case counts during 2015–2016 (Fig. 3). However, a few exceptions within these continents were found. For example, we estimated that 432 (332–582) ZIKV infections were imported in Shanghai, China, although not a single imported case was reported by the city. Similarly, an estimated number of 473 (322–734) ZIKV infections were imported in the Luanda spatial unit in Angola during 2015–2016 without being detected. As a by-product of the importation simulation model, we estimated a reporting index for each susceptible spatial unit, to quantify the probability of reporting a case with each importation (Additional File 6: Table S1). Note that for some countries presented in Fig. 3 and Additional File 6: Table S1, we were only able to find the total reported number of imported Zika cases at a country level (e.g. Australia, New Zealand). To facilitate the comparison of the reported and simulated data, each of these countries was treated as a single unit (only in Fig. 3 and Additional File 6: Table S1).

### Mapping the cryptic spread of ZIKV during 2015–2016

**Fig. 4:**
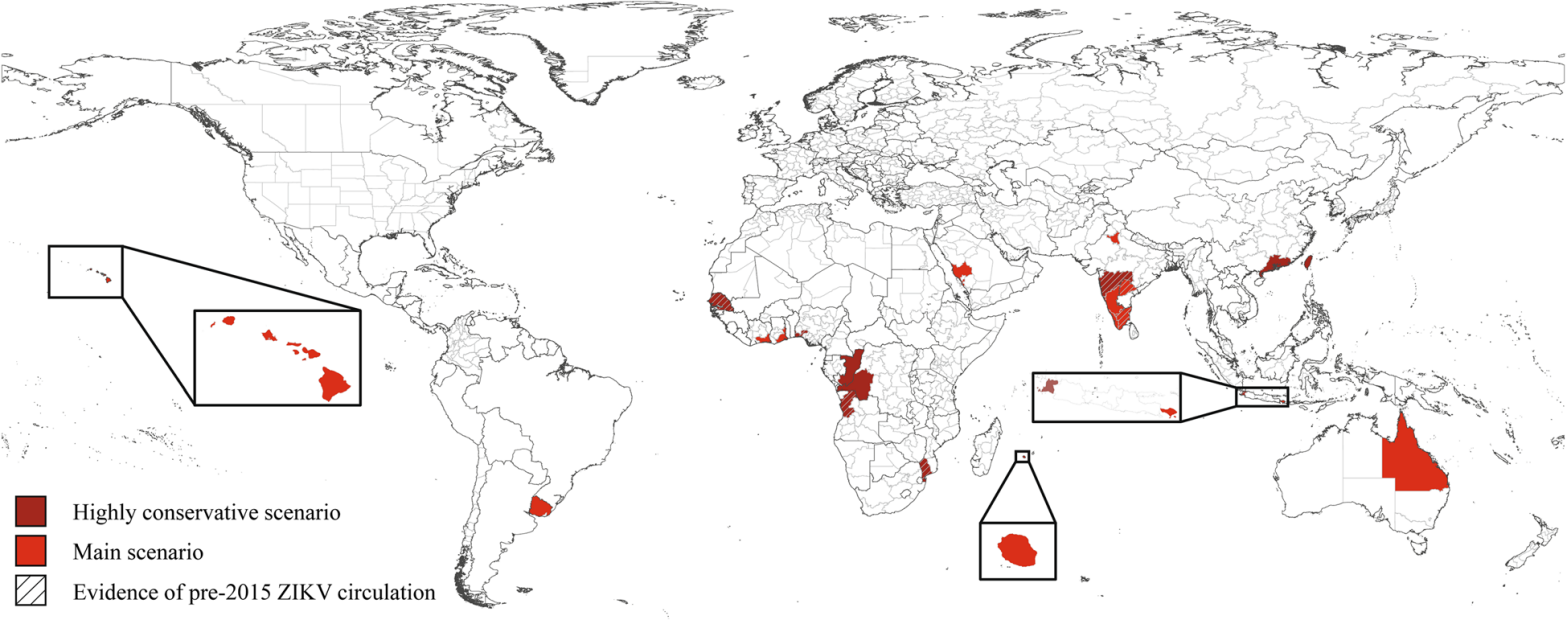
Susceptible spatial units with a high chance of cryptic ZIKV spread during 2015–2016 (shown in red). If evidence of historical ZIKV circulation prior to 2015 was found in any of these locations, the corresponding polygon is striped. Susceptible spatial units coloured in dark red refer to those that continue to have high potential for cryptic ZIKV spread during 2015–2016 under the highly conservative scenario (i.e. thermal restriction for ZIKV transmission being 26–29 °C)

A total of 24 susceptible spatial units were identified to have a high chance of cryptic ZIKV spread during 2015–2016 under our primary scenario, of which 21 (87.5%) are located in Asia or Africa (Table 2, Fig. 4). In particular, there were 10 susceptible spatial units estimated to have a significant risk of cryptic ZIKV spread during 2015–2016 even under the highly conservative scenario (i.e. thermal restriction for ZIKV transmission being 26–29 °C), namely, Luanda in Angola, Banten in Indonesia, Maharashtra in India, Lagos in Nigeria, Taiwan and Guangdong in China, Dakar in Senegal, Maputo in Mozambique, Kinshasa in Congo DRC, and Pool in Congo (Table 2, Fig. 4). Of all the 24 susceptible spatial units identified, we found 8 where there exists evidence of historical ZIKV circulation prior to 2015, although in most cases the serological data were collected many decades ago (Table 2) [51–56]. Refer to Additional File 7: Table S2 for the estimated probability that no onward spread of ZIKV occurred following importation during 2015–2016 in each susceptible spatial unit.

**Table 2:** Susceptible spatial units with a high chance of cryptic ZIKV spread during 2015–2016 (refer to Additional File 8: Table S3 for the list of first-level country subdivisions belonging to each susceptible spatial unit)

Country	Spatial unit name	Estimated probability ($\overline{\theta}$) that no onward spread of ZIKV had occurred during 2015–2016	Evidence of historical ZIKV circulation prior to 2015 (Y/N)
Angola	Luanda*	< 0.0001	Y [51]
Indonesia	Banten*	< 0.0001	Y [52]
India	Maharashtra*	< 0.0001	Y [53]
Nigeria	Lagos*	< 0.0001	Y [54]
China	Taiwan*	< 0.0001	N
China	Guangdong*	< 0.0001	N
India	Karnataka	0.0002	N
India	Tamil Nadu	0.0003	Y [53]
India	Delhi	0.0004	N
USA	Hawaii	0.0004	N
Senegal	Dakar*	0.0004	Y [55]
Uruguay	Canelones	0.0004	N
Réunion	Réunion	0.0004	N
Australia	Queensland	0.0005	N
Indonesia	Bali	0.0007	N
Mozambique	Maputo*	0.0007	Y [56]
Ghana	Greater Accra	0.0018	N
Congo DRC	Kinshasa*	0.0019	N
India	Kerala	0.0050	N
Saudi Arabia	Makkah	0.0115	N
India	Goa	0.0130	N
India	Telangana	0.0190	Y [53]
Congo	Pool*	0.0281	N
Côte d’Ivoire	Abidjan	0.0470	N^§^

*$\overline{\theta}$ remained lower than 0.05 even within the highly conservative scenario

^§^There was evidence of historical ZIKV circulation prior to 2015 in Côte d’Ivoire, but it was not clear whether any of the positive serum samples were collected from the Abidjan spatial unit or not

Note that some spatial units in our study may represent a group of subdivisions within a country. Within each of these spatial units, only one subdivision had nonzero incoming air passengers during 2015–2016 (see the “Methods” section for more details), which was assigned as the name for that spatial unit. For example, the susceptible spatial unit named “Luanda” in Table 2 contained four other subdivisions in Angola in addition to the Luanda subdivision itself. The list of all the first-level country subdivisions within each susceptible spatial unit was provided in Additional File 8: Table S3.

## Discussion

This study unveils the potential cryptic spread of ZIKV during 2015–2016 to further our understanding of the virus’s circulation worldwide. This was achieved via integrating heterogeneous data sources to model ZIKV’s transmissibility, importation, and onward spread, combined with the evidence of *Aedes*-borne disease transmission potential from the existing literature. Although ZIKV has received much less media attention ever since the epidemic waned, our results highlight the geographical areas where future studies may be needed to investigate if past or ongoing ZIKV circulation is present, in preparation for possible epidemics in the future.

Overall, the global distribution of the number of imported ZIKV infections based on our simulations was consistent with the importation risk estimates derived by Nah et al. [57], where countries in Western Europe and the Americas experienced a higher risk of importation in contrast to the majority of Asian and African countries. Several spatial units in Asia and Africa, however, were estimated to have a large number of imported ZIKV infections, such as Shanghai in China and Luanda in Angola, in agreement with the findings of Bogoch et al. [17]. Different from the previous studies, we directly estimated the absolute number of imported infections at a reasonably high spatial resolution, and our results were found to correlate very well with the imported case counts reported by different susceptible spatial units in the US.

To date, new evidence of ZIKV transmission continues to accumulate, which enables our estimated geographical extent of cryptic ZIKV spread to be partially validated. On 26 December 2016, the first Zika case in Angola was identified in a 14-year-old boy residing in Luanda, immediately after the initiation of ZIKV RT-PCR testing in the country [58]. Correspondingly, our model successfully detected autochthonous ZIKV transmission in Luanda, with the estimated probability of no indigenous cases occurring during 2015–2016 being the lowest (8.53 × 10^−31^) among all the 24 susceptible spatial units listed in Table 2. Note that we classified Luanda as a susceptible spatial unit in our study, since the first detected case was not announced until the early January of 2017 [59]. Besides Luanda, Tamil Nadu in India reported its first autochthonous Zika case in July 2017 [60]. Based on our model, 31 (19–46) ZIKV infections were imported in Tamil Nadu during 2015–2016, with the chance of no onward spread following these imported infections being low (0.0003). Thus, it was possible that ZIKV had already circulated silently in Tamil Nadu by the end of 2016, which later gave rise to the first reported case in 2017. Moreover, in a recent study published in October 2019, low ZIKV seroprevalence was observed in serum samples collected from Southern Taiwan at the end of 2015, providing evidence of possible undetected autochthonous transmission [61]. Overall, the abovementioned epidemiological evidence adds to the credibility of our results, and underscores the necessity of local investigation in the rest of the susceptible spatial units that were found to have a high potential of cryptic ZIKV spread during 2015–2016.

In Africa, information on the ZIKV seroprevalence and incidence remains limited and largely outdated, but recent evidence suggests that the virus has been silently spreading in the continent for at least two decades [55]. According to Herrera et al., of the 387 serum samples collected from febrile patients in Nigeria and Senegal during 1992–2016, 6.2% were positive for IgM to ZIKV and negative for dengue reactivity, with four samples from which ZIKV envelope was amplified [55]. Thus, there are at least two plausible interpretations for the 9 susceptible spatial units in Africa listed in Table 2. These spatial units could have indeed experienced ZIKV spread during 2015–2016, which was undetected due to limited surveillance capacity. Alternatively, the level of herd immunity prior to 2015 may have been higher than currently known (due to paucity of serological data), thereby successfully preventing ZIKV transmission during 2015–2016. In either case, a significant knowledge gap remains to be filled, and this is particularly critical in light of the past or current spread of dengue, Chikungunya, or yellow fever viruses in these spatial units, which shows that the future risk of ZIKV epidemics should not be ignored. For example, a dengue outbreak occurred in Abidjan city, Côte d’Ivoire in 2019, where a total of 1776 suspected dengue cases were reported between 1 January and 25 June [62]. Recent entomological investigations carried out in Abidjan also revealed a very high larval index of *Ae. aegypti*, the primary vector species of ZIKV [63]. Elsewhere, a cross-sectional study conducted between November 2015 and June 2016 in Kinshasa, Democratic Republic of Congo, found that 30.2% and 26.4% of the participants had experienced past dengue and Chikungunya infections respectively [64]. Although no acute ZIKV infections were found, of note is that much fewer blood samples (*n* = 80) were tested for ZIKV, with no urine samples collected and only RT-PCR performed [64]. Thus, the possibility of low-level cryptic transmission of ZIKV in Kinshasa remains to be investigated.

In South Asia, the presence of ZIKV antibodies in humans was first discovered in India in 1952 [53]. More recently, India reported its first three laboratory-confirmed indigenous Zika cases in Gujarat on 15 May 2017, months after they were detected [65]. Subsequently, another case was found in Tamil Nadu during the same year [60]. After testing over 35,000 febrile illness serum samples, the Indian government had only identified the aforementioned four cases by 2017, indicating a very low level of ZIKV circulation [66]. Interestingly, we have found seven spatial units in the country with a high potential of cryptic ZIKV spread during 2015–2016, and these included Tamil Nadu but not Gujarat. Based on the representative partial sequences, the virus from Gujarat seems to belong to an old Asian strain, in contrast to the ZIKV in Tamil Nadu, which was classified as the then-current Asian outbreak strain [67]. It is thus possible that the unreported cases in Gujarat were not imported from any of the donor spatial units in our study and hence were not detected by our model. The four Zika cases reported in 2017, together with the following 2018 outbreaks in Rajasthan and Madhya Pradesh [67], demonstrate the importance of strengthening ZIKV surveillance in other states as well, especially the ones where we found a high potential for cryptic spread of ZIKV. Given the considerably high intensity of dengue transmission in India [68], any low-level spread of ZIKV could be easily undetected due to misdiagnosis, until the virus causes a major outbreak possibly in the future.

In Southeast Asia, data on ZIKV epidemiology is scarce despite the long-term circulation of the virus [7]. Our study has identified two provinces in Indonesia—Bali and Banten—where cryptic ZIKV spread was likely to have occurred during 2015–2016. The earliest serological evidence of ZIKV transmission within the country was obtained in Central Java and Lombok around the 1970s [69, 70], and the virus was found to remain circulating in the early 2010s [52]. Specifically, among the 662 samples collected from healthy children aged 1–4 years in 2014, 9.1% were ZIKV-seropositive, who came from 11 provinces including Banten but not Bali [52]. The total number of serum samples obtained from these two provinces was limited (67 combined) [52], and the level of ZIKV immunity among the general population was still unclear. Besides Southeast Asia, we also found two spatial units in East Asia that may require future risk assessment, namely Guangdong and Taiwan. Both spatial units have reported dengue outbreaks within the past few years [71, 72], as well as imported ZIKV infections as recently as the early 2020 [73, 74]. These, combined with the latest discovery of low ZIKV seroprevalence in southern Taiwan and a neighbouring province of Guangdong [61, 75], suggest that the potential risk of future ZIKV outbreaks should not be overlooked.

In West Asia, our results highlight only one spatial unit that requires special attention, namely Makkah. In 2009, a dengue epidemic occurred in the city, with a 20-fold increase in the incidence rate compared with the year before, and the disease became endemic since then [76], suggesting the local environment could also be suitable for ZIKV transmission. We estimated that at least 22 (12–36) ZIKV infections were imported in Makkah during 2015–2016, with a very low chance of no onward transmission (0.012). Notably, the city receives ~ 200,000 pilgrims from Indonesia annually [77], where ZIKV is presumed to be endemic [52], thus facing a potentially ongoing risk of ZIKV importation every year. To date, no indigenous Zika cases in Makkah have been reported, but serological data indicate that the virus may have circulated elsewhere within the country. In a recent study involving 410 asymptomatic pregnant women in Najran, 24 tested positive for anti-ZIKV IgM and 52 were anti-ZIKV IgG positive, none of whom had travelled abroad [78]. Although no acute infections were detected using RT-PCR and cross-reactivity with dengue virus could not be excluded, the study points to the possibility of silent ZIKV spread in Saudi Arabia, which needs to be further assessed by larger studies at the nationwide level [78].

Outside of Africa and Asia, we found high potential for cryptic ZIKV spread during 2015–2016 in the following spatial units: Canelones in Uruguay (which covers the vast majority of the country), Hawaii in the USA, and Queensland in Australia. Being one of the very few countries in the Americas without any ZIKV transmission reported to date [7], Uruguay also did not find any autochthonous dengue cases until an outbreak which occurred in the early 2016 [79]. The high estimated risk of cryptic ZIKV spread in the Canelones spatial unit during 2015–2016 was likely to be attributed to the very large number of imported ZIKV infections, which we estimated to be 2043 (1537–2913). In contrast, both Hawaii and Queensland were estimated to have much fewer imported ZIKV infections but meanwhile higher environmental suitability for *Ae. aegypti*, with multiple historical dengue epidemics reported to date [80, 81], and hence may face a higher risk of local ZIKV outbreak in the future.

Zika has not gone away. It is thought that low-level transmission is still ongoing in many countries in the world [7]. The identification of locations with likely cryptic spread of ZIKV during the 2015–2016 global epidemic can have significant public health implications, as evidenced by the previous silent circulation of the virus in northeast Brazil that eventually caused a huge epidemic nationally and internationally [82]. Even in spatial units where winter months exist (e.g. Guangdong), ZIKV can be transmitted vertically in *Ae. aegypti* as a means of survival under adverse conditions, and thus the epidemiological importance of cryptic ZIKV spread in these areas should not be discounted [83]. In addition, improving knowledge about global ZIKV spread could also contribute to a better understanding of dengue epidemiology, given the immune interactions between these closely-related flaviviruses as demonstrated by a recent study [84].

Our study must be interpreted in light of the following limitations. For each susceptible spatial unit, we were unable to quantify the population-level immunity immediately prior to the 2015–2016 global ZIKV epidemic, largely owing to the paucity of updated serological data, which also include antibody titre data that are needed to account for the effect of pre-existing dengue immunity on the susceptibility to ZIKV infection in each location [85]. This is compounded by the complex effect of vector-virus genetic interactions on ZIKV transmissibility [86], which is still poorly understood in general and outside the scope of our study. In addition, we did not account for land or sea transport in our analysis, and assumed their impact on our estimates to be limited given that most ZIKV infections that were imported from *donor* to *susceptible* spatial units involved air travel, although short-range commuting flows could play a more important role in the epidemic invasion path once the virus was introduced to a new country or continent [87]. We also did not consider importation caused by infected passengers in transit who entered a country as short-term visitors before reaching their final destinations, due to the generally short LOS presumed for this relatively small number of individuals. Besides, our study did not account for the impact of weather variables such as rainfall on the seasonal variation in vector-to-host ratios, which is challenging to model on a global scale and can be further explored in future work.

Finally, we relied on only the locations with reported indigenous Zika cases as sources of importation, and hence, both the estimated number of imported infections and the geographical extent of cryptic ZIKV spread during 2015–2016 were conservative. In other words, it is not possible to reveal all the locations at a high risk of cryptic ZIKV spread in a single modelling study, and the findings generated in this study were conditional on the currently available data only. For instance, it is thought that ZIKV may be endemic in many parts of Southeast Asia, but epidemiological data are seriously lacking [7], thereby hindering a more comprehensive analysis of ZIKV spread within the region. Nonetheless, this study has made an important contribution to narrowing the knowledge gap of the global ZIKV epidemiology and has also laid a foundation for future studies that attempt to further explore this important topic. For example, once previously undetected circulation of ZIKV is confirmed by serological/RT-PCR testing at locations that we estimated as having a high potential of cryptic ZIKV spread, future modelling studies can then aim to identify geographical areas with (1) high connectivity to the aforementioned locations and (2) local evidence of *Aedes*-borne disease transmission potential, to further narrow this key knowledge gap.

## Conclusions

In conclusion, our study has provided valuable insights into the potentially high-risk locations for cryptic ZIKV circulation during the 2015–2016 pandemic. Enhanced surveillance is recommended in these geographical areas to mitigate the risk of future epidemics—locally, nationally, and even globally—given the world is increasingly vulnerable to pandemic threats due to the expansion of air traffic networks. In the context of the growing importance of enhanced vigilance and epidemic preparedness in today’s world, our modelling framework can also be adapted to identify areas with likely unknown spread of other emerging vector-borne diseases, which has important implications for public health readiness especially in resource-limited settings.

## Supplementary information

**Additional file 1.** Supporting information.

**Additional file 2 **: **Data S1.** Weekly number of reported autochthonous Zika cases by each country or subdivision during 2015–2016.

**Additional file 3.** : R Code.

**Additional file 4 **: **Fig. S1.** Median estimate of ZIKV *R*_0_ for each spatial unit obtained from the global risk model at Eweeks (A) 2, (B) 12, (C) 22, (D) 32, (E) 42, and (F) 52 in 2016 respectively. Note that these estimates did not incorporate local evidence of *Aedes*-borne disease transmission potential or thermal restrictions for ZIKV transmission under different scenarios. Additional adjustment steps were implemented in the analysis of onward ZIKV spread to minimize false positive rates (Refer to the Methods section for more details).

**Additional file 5 **: **Fig. S2.** Estimated ZIKV *R*_0_ presented as a function of temperature and vector-to-host ratio. For demonstration purposes, we assumed an equal vector-to-host ratio for *Ae. aegypti* and *Ae. albopictus*, and the y-axis value refers to the vector-to-host ratio for *each* species. Note that these estimates were preliminary results only, which did not incorporate the thermal restrictions for ZIKV transmission under different scenarios. An additional adjustment step was implemented in the analysis of onward ZIKV spread to minimize false positive rates (Refer to the Methods section for more details).

**Additional file 6 **: **Table S1**. Reported / simulated total number of Zika cases / ZIKV infections imported in each susceptible spatial unit during 2015–2016, together with the estimated reporting index.

**Additional file 7 **: **Table S2**. Estimated probability that no onward spread of ZIKV occurred following importation during 2015–2016 for each susceptible spatial unit.

**Additional file 8 **: **Table S3**. List of first-level country subdivisions belonging to each susceptible spatial unit.

## Data Availability

Reported autochthonous Zika case data during 2015–2016 have been included within Additional File 2: Data S1. Raster maps of the environmental suitability for *Aedes aegypti* and *Aedes albopictus* are available from the author upon reasonable request. R code has been included within Additional File 3.

## Abbreviations

Eweek: Epidemiological week
LOS: Length of stay
OAG: Official Airline Guide
PAHO: Pan American Health Organization
SEDAC: Socioeconomic Data and Applications Centre
UNWTO: United Nations World Tourism Organization
ZIKV: Zika virus
