# Supplementary material for: Mapping the cryptic spread of the 2015–2016 global Zika virus epidemic
Source: BMC Med. 2020 Dec 17;18:399. doi: 10.1186/s12916-020-01845-x (PMC7744256; doi:10.1186/s12916-020-01845-x)

Mapping the cryptic spread of the 2015–6 global Zika virus epidemic

Haoyang Sun[1, §] (B.Sc.), Borame L Dickens[1] (Ph.D.), Mark Jit [2, 3] (Ph.D.), Alex R Cook [1, *, §] (Ph.D.), L Roman Carrasco [4, *](Ph.D.)

[1] Saw Swee Hock School of Public Health, National University of Singapore, 12 Science Drive 2, Singapore 117549, Republic of Singapore

[2] Department of Infectious Disease Epidemiology, London School of Hygiene and Tropical Medicine, Keppel Street, London, WC1E 7HT, United Kingdom

[3] Modelling and Economics Unit, Public Health England, London, United Kingdom

[4] Department of Biological Sciences, National University of Singapore, 14 Science Drive 4, Singapore 117543, Republic of Singapore

[*] Contributed equally.

[§] Correspondence to:

Haoyang Sun. Saw Swee Hock School of Public Health, National University of Singapore, 12 Science Drive 2, Singapore 117549, Republic of Singapore. Email: ephsunh@nus.edu.sg.

Alex R Cook. Saw Swee Hock School of Public Health, National University of Singapore, 12 Science Drive 2, Singapore 117549, Republic of Singapore. Email: ephcar@nus.edu.sg.

Supporting information

1. Additional information on the collection and pre-processing of data

1.1 Reported autochthonous Zika case data:

[Group 1]: American Samoa, Papua New Guinea, Thailand.

Cases were reported monthly at a country level. We assumed the daily case count to be uniform within each month to calculate the weekly incidence. If the monthly number of reported cases was below four, we randomly assigned each case to a Eweek within that month.

[Group 2]: Anguilla, Antigua and Barbuda, Argentina, Aruba, Bahamas, Barbados, Belize, Bolivia, British Virgin Islands, Cayman Islands, Costa Rica, Cuba, Curacao, Dominica, Dominican Republic, Ecuador, El Salvador, French Guiana, Grenada, Guadeloupe, Guatemala, Guyana, Haiti, Honduras, Jamaica, Martinique, Mexico, Nicaragua, Panama, Paraguay, Peru, Puerto Rico, Saint Barthelemy, Saint Kitts and Nevis, Saint Vincent and the Grenadines, Suriname, Trinidad and Tobago, Turks and Caicos Islands, United States, US Virgin Islands, Venezuela.

Weekly autochthonous case counts were published by the Pan American Health Organization (PAHO) as bar charts, which were digitized by the Anderson Lab and made publicly available. Except the United States, where data were available for each state (Florida and Texas), the rest of the countries in Group 2 had incidence data published at a country level only.

[Group 3]: Bonaire, Sint Eustatius, and Saba; Cabo Verde; Micronesia; Montserrat; Saint Martin; Samoa; Sint Maarten; Tonga.

We digitized the weekly country-level autochthonous case counts from published bar charts, except for Tonga, for which data were directly obtained from the table published by the ReliefWeb.

[Group 4]: Brazil

Weekly number of autochthonous cases reported by Brazil during 2016 were digitized by the Anderson Lab based on bar charts published by the PAHO. We manually digitized the weekly number of notified Zika cases in Brazil during 2015 published by Lourenço et al. (2018), since these data were not released by the PAHO.

Ideally, we would want to obtain case data reported at a subdivision level, given that Brazil reported the largest number of autochthonous Zika cases during 2015–6 and these cases were not evenly distributed across space. We were unable to obtain the subdivision-level data however and therefore ran the analysis at a country level.

[Group 5]: Colombia

For each first-level country subdivision except San Andrés, weekly cumulative case counts between Eweek 37 and Eweek 52 of 2016 were obtained from the Colombian National Institute of Health's website, and each time series was then differenced to derive the weekly case counts. The weekly ZIKV incidence data reported prior to Eweek 38 of 2016 were collected and made publicly available by Siraj et al. (2018).

For San Andrés, weekly incidence data were not published by Siraj et al. (2018). Hence, we obtained daily incidence data prior to Eweek 5 of 2016 from Rojas et al. (2016), which were subsequently aggregated within each Eweek. As before, weekly incidence data from Eweek 5 of 2016 onwards were derived from the weekly cumulative case counts published by the Colombian National Institute of Health.

[Group 6] Fiji, Maldives, Philippines, Solomon Islands, Vanuatu, Vietnam.

These countries were excluded from our analyses, because either the autochthonous case counts were not publicly available, or the time of each reported case could not even be narrowed down to a specific month. Overall, the number of autochthonous Zika cases reported by these countries only accounted for less than 0.09% of the total case count reported worldwide during 2015–6.

[Group 7]: Guinea-Bissau, Indonesia, Laos, Malaysia, Myanmar, New Caledonia, Palau.

The total number of autochthonous cases reported by each of these countries during 2015–6 was relatively small (less than ten per country). The Eweek during which each case was reported can be found using HealthMap. We located each autochthonous case reported by Indonesia, Malaysia, and Myanmar to a first-level country subdivision, and data for the rest of the countries were publicly available at a country level only.

[Group 8]: Marshall Islands, Singapore.

We digitized the daily country-level autochthonous case counts from published bar charts, which were subsequently aggregated within each Eweek.

[Group 9] Saint Lucia

The PAHO published the total number of autochthonous Zika cases reported at a country level by the end of 2016, and the time when the first autochthonous case was identified. Since the total number of cases was reasonably large (872), we did not exclude Saint Lucia from our analysis, and assumed the shape of the epidemiological curve (post-standardization) was equal to that of Saint Vincent and the Grenadines, which is situated closed to Saint Lucia.

1.2 Merging of subdivisions

For each first-level country subdivision s with no incoming air passengers based on the 2015–6 OAG data, we performed an estimation of the most likely airport a^* (within the same country) that the population of subdivision s would rely on when they returned home. In the equation below, the distance between the population-weighted centroid coordinates of subdivision s and an airport a (denoted by $d(a,s)$) was adjusted by the total number of incoming air passengers during 2015–6 (v_a) using Holling's type 2 function, so that major airports would not be overemphasized. We used the notation "a within c_s" to restrict ourselves to all airports a within the country c_s, to which subdivision s belonged (similarly for "a' within c_s"):

$$a^* = \mathrm{argmin}_{a \text{ within } c_s} \frac{d(a,s)}{\sigma(a)}$$

$$\sigma(a) = \mathrm{max}_{a' \text{within } c_s}(v_{a'}) \cdot \frac{v_a}{v_a + \mathrm{median}_{a' \text{within } c_s}(v_{a'})}$$

Consequently, subdivision $\boldsymbol{s}$ was merged with the one where airport a^* was located, and they were modelled as a single unit from then on. However, if the reported autochthonous cases cannot be located to the subdivision level, the entire country was treated as a single unit of analysis. Hence, the spatial unit of analysis in our study can be a single first-level country subdivision, a combination of subdivisions, or an entire country (hereinafter referred to as "spatial unit").

1.3 Countries or territories with zero incoming air passenger during 2015–6, and hence were excluded from the analyses.

Andorra, Bouvet Island, British Indian Ocean Territory, Clipperton, French Southern Territories, Heard Island and McDonald Islands, Liechtenstein, Palestinian Territory, Pitcairn, Saint Helena, San Marino, South Georgia, Tokelau, Vatican City.

2. Additional information on the global risk model

2.1 Model validation

We took the list of R_0 estimates that were obtained by Armstrong et al. (2020) via a systematic search within Web of Science and PubMed, and applied the following selection criteria:

1) The R_0 value was derived by estimating the early growth rate of the epidemic based on the reported case data (i.e. using a simple exponential growth model, or Richards' growth model).

In other words, we did not include estimates derived from mechanistic models, which could vary substantially in terms of the functional forms as well as the input parameter values used. In fact, even for the same location and time window, there can be very different literature estimates of R_0 obtained by these models, where the differences in model specifications played an important role. This is not to say that these model results were all invalid, but the complex nature of the models has made it challenging to standardize the results, as well as to examine the validity of the methods given the large number of studies included in the list. Hence, we only included estimates that were directly driven by the reported case data as described in the beginning.

2) The time period of the reported case data that were used to estimate R_0 was clearly specified.

This would allow us to average our R_0 estimates across the corresponding time window to be compared with the literature estimate.

3) The spatial resolution of the location of the reported case data that were used to estimate R_0 should not be higher than the spatial unit of analysis in our study.

4) The width of the uncertainty interval of the R_0 estimate should not exceed 10.

2.2 Visualizations of the global risk model outputs

Figure S1: Median estimate of ZIKV R_0 for each spatial unit obtained from the global risk model at Eweeks (A) 2, (B) 12, (C) 22, (D) 32, (E) 42, and (F) 52 in 2016 respectively.

Note that these estimates did not incorporate local evidence of *Aedes*-borne disease transmission potential or thermal restrictions for ZIKV transmission under different scenarios. Additional adjustment steps were implemented in the analysis of onward ZIKV spread to minimize false positive rates (Refer to the Methods section for more details).

[See Figure S1.tif in the attachment]

Figure S2: Estimated ZIKV R_0 presented as a function of temperature and vector-to-host ratio*.

For demonstration purposes, we assumed an equal vector-to-host ratio for *Ae. aegypti* and *Ae. albopictus*, and the y-axis value refers to the vector-to-host ratio for *each* species. Note that these estimates were preliminary results only, which did not incorporate the thermal restrictions for ZIKV transmission under different scenarios. An additional adjustment step was implemented in the analysis of onward ZIKV spread to minimize false positive rates (Refer to the Methods section for more details).

* The 2.5th, 25th, 50th, 75th, and 97.5th percentiles of the estimated vector-to-host ratios for all the spatial units were: 0.00, 0.71, 2.00, 6.41, and 10.70 respectively for *Ae. aegypti* and 0.00, 0.63, 1.63, 5.52, and 10.17 respectively for *Ae. albopictus*.

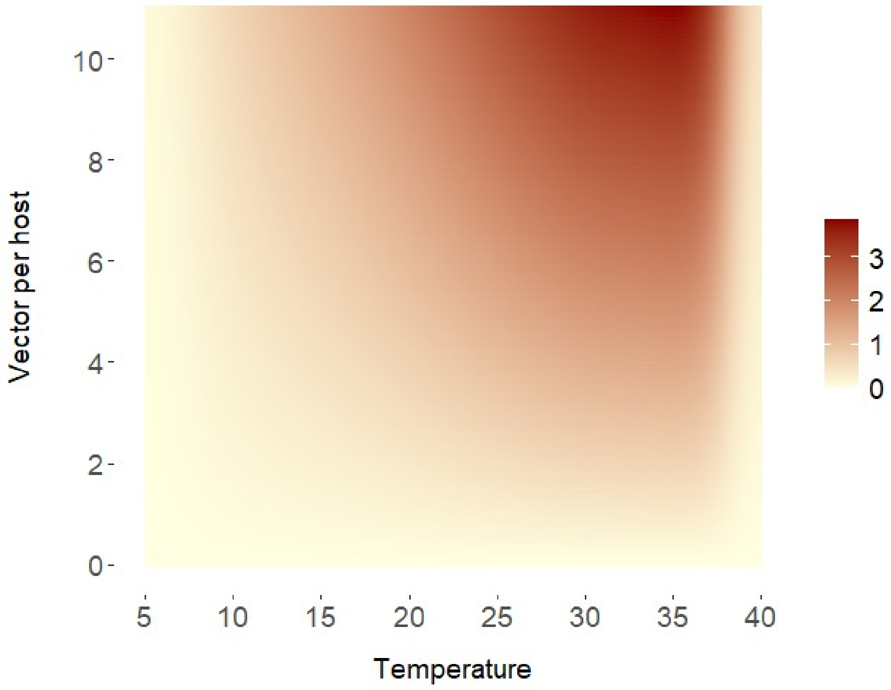

Supplement: Supplementary file 1 — Additional file 1. Supporting information. [file 12916_2020_1845_MOESM1_ESM.pdf]
