# Supplementary figures and images for: Mapping the cryptic spread of the 2015–2016 global Zika virus epidemic
Source: BMC Med. 2020 Dec 17;18:399. doi: 10.1186/s12916-020-01845-x (PMC7744256; doi:10.1186/s12916-020-01845-x)

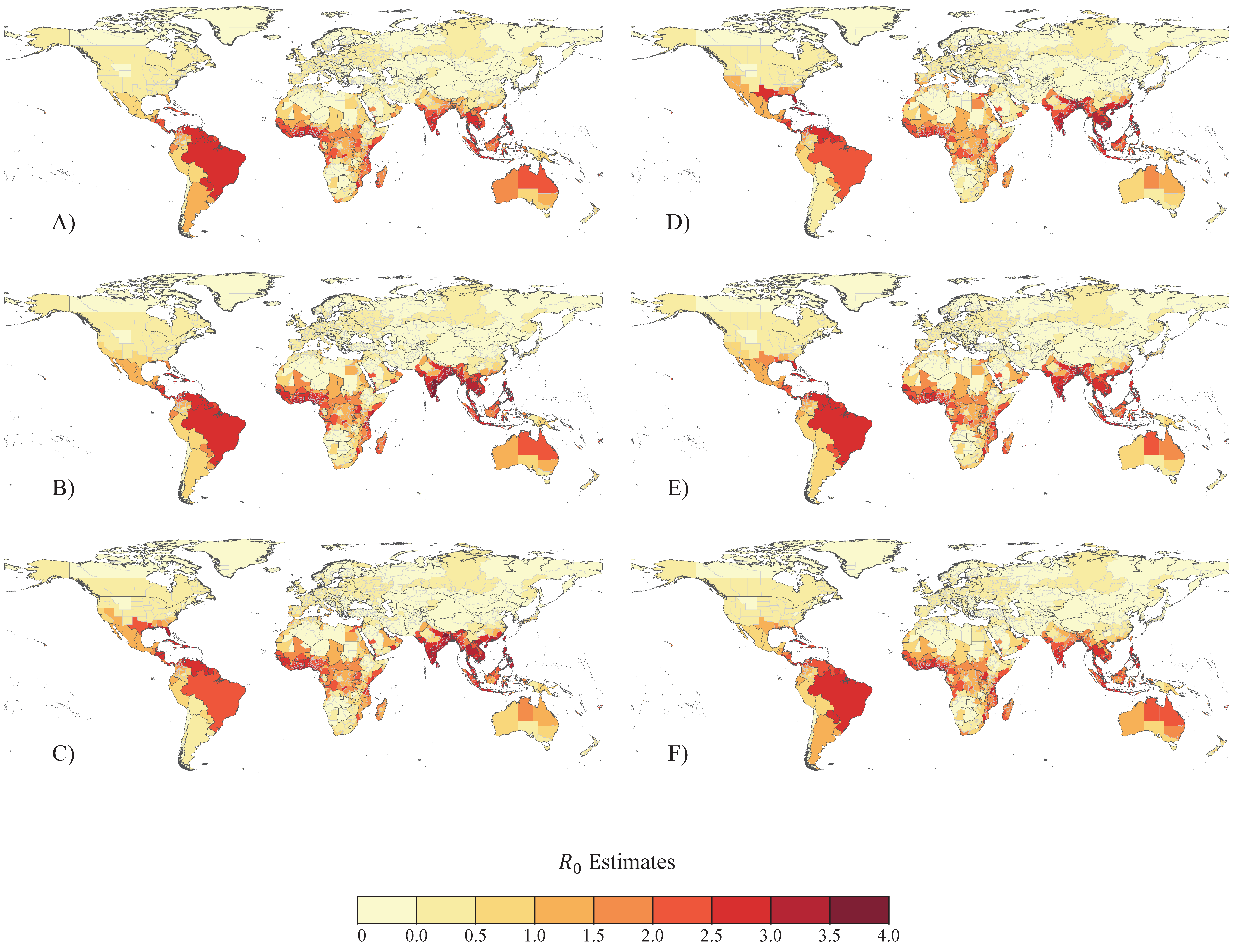

Supplement: Supplementary file 4 — Additional file 4 : Fig. S1. Median estimate of ZIKV R0 for each spatial unit obtained from the global risk model at Eweeks (A) 2, (B) 12, (C) 22, (D) 32, (E) 42, and (F) 52 in 2016 respectively. Note that these estimates did not incorporate local evidence of Aedes-borne disease transmission potential or thermal restrictions for ZIKV transmission under different scenarios. Additional adjustment steps were implemented in the analysis of onward ZIKV spread to minimize false positive rates (Refer to the Methods section for more details). [file 12916_2020_1845_MOESM4_ESM.tif]

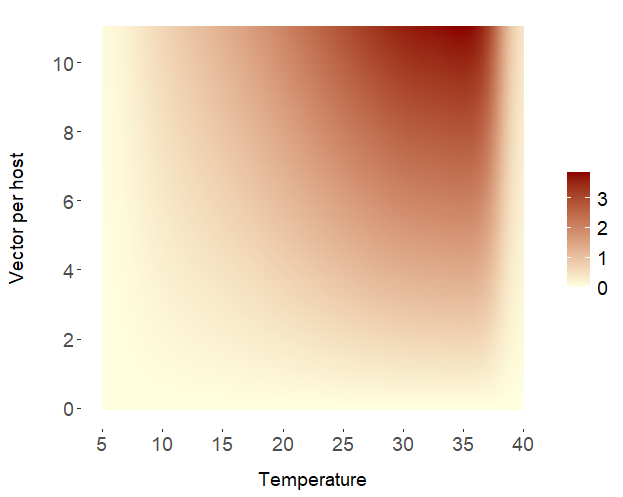

Supplement: Supplementary file 5 — Additional file 5 : Fig. S2. Estimated ZIKV R0 presented as a function of temperature and vector-to-host ratio. For demonstration purposes, we assumed an equal vector-to-host ratio for Ae. aegypti and Ae. albopictus, and the y-axis value refers to the vector-to-host ratio for each species. Note that these estimates were preliminary results only, which did not incorporate the thermal restrictions for ZIKV transmission under different scenarios. An additional adjustment step was implemented in the analysis of onward ZIKV spread to minimize false positive rates (Refer to the Methods section for more details). [file 12916_2020_1845_MOESM5_ESM.png]
